# Relationship between C-reactive protein-to-albumin ratio and new-onset coronary artery disease and its severity in different glucose metabolic states: A retrospective cohort study

**DOI:** 10.1097/MD.0000000000049238

**Published:** 2026-06-05

**Authors:** Sisi Han, Liangbo Chen, Hongyun Shu, Guojun Zhao, Henghui Zhang, Jianhong Li, Weiping Qiu, Jingsong Cheng, Guihua Zheng, Qiaowen Li

**Affiliations:** a Department of Cardiovascsular Medicine, Affiliated Qingyuan Hospital, Guangzhou Medical University, Qingyuan People’s Hospital, Guangdong, China; b Department of Cardiovascular Medicine, Affiliated Hospital of Guangdong Medical University, Zhanjiang, China; c Institute of Gerontology, Guangzhou Geriatric Hospital, Guangzhou Medical University, Guangzhou, China; d Department of Urology, The Fifth Affiliated Hospital of Guangzhou Medical University, Guangzhou, Guangdong, China.

**Keywords:** C-reactive protein-to-albumin ratio, coronary artery disease, coronary artery disease severity, glucose metabolic states, inflammation

## Abstract

The C-reactive protein-to-albumin ratio (CAR) integrates inflammatory and nutritional status.The study aimed to explore the potential connection between CAR and new-onset coronary artery disease (CAD) presence across varying glucose levels and to determine its correlation with the severity of coronary atherosclerosis. The retrospective cohort comprised 1489 individuals receiving coronary angiography for the first time. Using glycemic status criteria, the cohort was divided into 3 subgroups: normoglycemic (NGR, n = 255), prediabetic mellitus (Pre-DM, n = 617), and diabetic mellitus (DM, n = 618). CAR quartiles were analyzed for associations with CAD presence and Gensini score using multivariable logistic regression. And the predictive utility of the CAR for CAD was determined via analysis of the receiver operating characteristic curve. After multivariate adjustment, elevated CAR independently predicted new-onset CAD risk in the overall cohort. Stratified by glucose metabolism, the highest CAR quartile (Q4) significantly increased CAD risk in NGR (odds ratio [OR] = 4.25, *P* < .05) and DM (OR = 2.29, *P* < .05), while the association in Pre-DM attenuated post-adjustment. Critically, CAR robustly correlated with CAD severity in Pre-DM and DM, most pronounced in DM. CAR predicted CAD overall (area under the curve = 0.638, 95% confidence interval: 0.605–0.672), outperforming isolated CRP or albumin. CAR serves as an independent predictor for new-onset CAD and coronary lesion severity, with predictive efficacy modulated by glucose metabolic states. Its association with CAD risk is pronounced in NGR and DM, while Pre-DM exhibits attenuated significance.

## 1. Introduction

Pathologically characterized by coronary arterial stenosis, coronary artery disease (CAD) constitutes a principal determinant of worldwide mortality and disability.^[1]^ The main mechanism of CAD development is atherosclerosis in the coronary arteries, leading to narrowing or blockage, reducing coronary perfusion and impaired oxygen availability, precipitating ischemic cardiomyocyte damage. In the early stages of CAD pathogenesis, disease severity and patient outcomes are predicted by inflammation-mediated endothelial impairment, blood flow abnormalities, and lipid dysregulation.^[2]^ Additionally, research indicated that different states of glucose metabolism, such as impaired glucose tolerance and type 2 diabetes mellitus (T2DM), were traditional risk factors for cardiovascular diseases, playing a crucial role in the development of advanced CAD as well as systemic atherosclerosis.^[3]^

C-reactive protein (CRP), the most extensively validated inflammatory mediator, enables the objective stratification of systemic inflammatory burden. The pathophysiological cascade from CRP to inflammation, then to atherosclerosis, and ultimately to CAD is well established: CRP drives a pro-inflammatory state that promotes endothelial dysfunction, accelerates plaque formation, and increases plaque vulnerability. In the context of T2DM, this cascade is both amplified and accelerated. Chronic hyperglycemia and insulin resistance induce a sustained low-grade inflammatory state characterized by elevated CRP levels, which in turn exacerbates endothelial dysfunction and fuels the progression of coronary atherosclerosis.^[4]^ Some researchers suggested that the correlation with CRP and incident T2DM was more prominent in elderly patients. CRP, in conjunction with obesity and hypertension, was linked to an increased risk of T2DM.^[1,4]^ Albumin (ALB), a hepatically synthesized plasma protein, serves as a dual-functional indicator for both nutritional status assessment and clinical outcome prediction.^[5]^ In pathological conditions such as chronic inflammation and diabetes, albumin synthesis is downregulated at the transcriptional level by pro-inflammatory cytokines, including interleukin-6 and tumor necrosis factor-alpha, while its catabolism is accelerated under oxidative stress and insulin-resistant states.^[6]^ Research indicated that serum ALB levels exhibited an inverse association with the incidence of diabetes and associated capillary dysfunction.^[7]^ Furthermore, ALB levels are significantly inversely correlated with CAD mortality risk, reduced anti-inflammatory activity, and attenuated oxidative stress. ALB demonstrates strong prognostic capacity in CAD and peripheral arterial disease patients. Notably, the predictive power of ALB significantly improves through combinatorial assessment with other risk biomarkers, such as CRP and serum uric acid.^[8,9]^ Collectively, both CRP and ALB not only serve as predictive biomarkers for CAD severity, but also exhibit intimate connections with dysglycemia.

Incorporation of ALB alongside CRP, the C-reactive protein-to-Albumin ratio (CAR) demonstrates the inflammatory and nutritional status of the body. A retrospective study identified CAR as an independent predictor of non-reperfusion in patients with acute ST-segment elevation myocardial infarction after undergoing percutaneous coronary intervention,^[10]^ suggesting that the CAR level and the severity of coronary artery stenosis may exist between the potential associated. Given the established connections of both CRP and ALB with dysglycemia and CAD, yet the undefined role of CAR across glycemic strata and its precise relationship with coronary lesion complexity, this study aims to explore the potential connection between CAR and CAD presence across varying glucose levels and to determine its correlation with the severity of coronary atherosclerosis.

## 2. Methods

### 2.1. Ethical approval

The research ethics board of the Affiliated Qingyuan Hospital (Qingyuan People’s Hospital) of Guangzhou Medical University granted formal authorization (IRB-2025-057) for all study procedures. The requirement of informed consent was waived due to its retrospective nature.

### 2.2. Study participants

**Figure 1.**
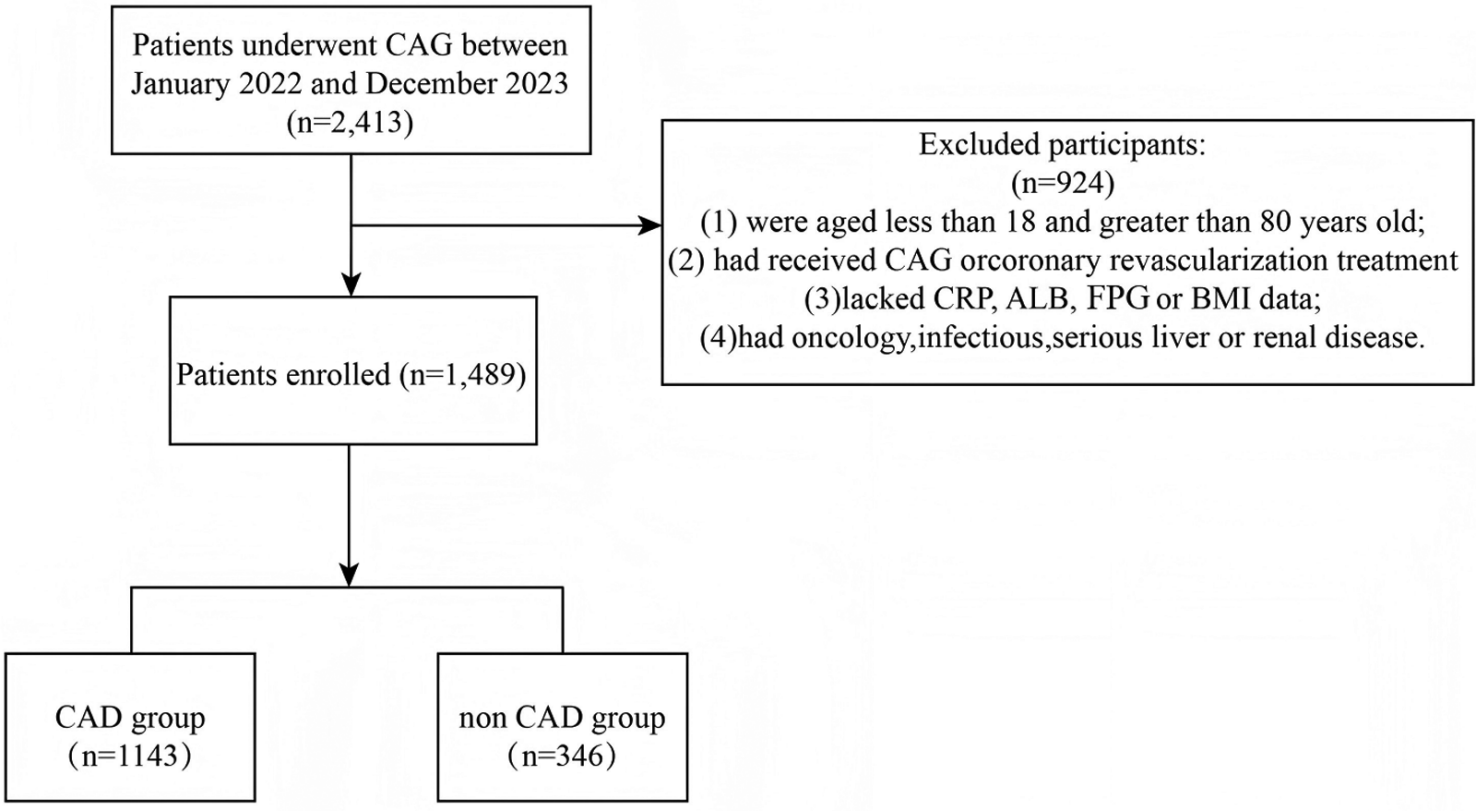
Flow chart of subject recruitment.CAG = coronary angiography, CAD = coronary artery disease, CRP = C-reactive protein, ALB = albumin, BMI = body mass index, FPG = fasting plasma glucose

This retrospective study included 2413 patients who underwent coronary angiography (CAG) procedures and were hospitalized at the Qingyuan Hospital, affiliated with Guangzhou Medical University, from January 2022 to December 2023. Inclusion criteria were clinical presentation of suspected stable ischemic heart disease (chest pain or dyspnea) necessitating first-time diagnostic coronary angiography (CAG); availability of complete laboratory data. Exclusion criteria comprised: were aged <18 and >80 years old; had received CAG orcoronary revascularization treatment; lacked CRP, ALB, fasting plasma glucose (FPG) or BMI data; had oncology, infectious, severe hepatic dysfunction (alanine aminotransferase > 3 × upper limit of normal) or renal insufficiency (estimated glomerular filtration rate < 30 mL/min/1.73m^2^). The study cohort comprised 1489 eligible participants, with the screening and enrollment process detailed in Figure 1. Participants were then grouped based on CAR quartiles.

### 2.3. Data source and collection

The clinical dataset was assembled by qualified healthcare practitioners using an automated electronic health record platform. This repository contained essential patient information, such as baseline demographic profiles, medical history, laboratory blood test findings, and associated diagnostic imaging reports. The demographic variables comprised age, sex, body weight, stature, systolic and diastolic blood pressure readings, as well as self-reported tobacco and alcohol use behaviors. Clinical history comprises a medical record of hypertension and diabetes, as well as medication treatment status. Medication treatment involves antihypertensive drugs, antidiabetic drugs, and antiplatelet drugs.

A fasting venous blood sample was collected by professionals in the morning. All biochemical parameters were measured uniformly by the Department of Clinical Laboratory of the Affiliated Qingyuan Hospital (Qingyuan People’s Hospital) of Guangzhou Medical University. All biochemical parameters, including liver function tests (ALT, AST, TBIL), lipids, glucose, and albumin, were measured on a Beckman AU5800 analyzer (Beckman Coulter, USA) using enzymatic kits. CRP was measured by immunoturbidimetric assay (Denka Seiken, Japan). The laboratory follows ISO 15189:2022 with daily quality control.

Diagnostic coronary angiography was conducted via transradial or transfemoral arterial access utilizing catheterization techniques. The advanced angiographic imaging system employed demonstrates broad clinical applicability, enabling precise detection of various coronary artery pathologies.

### 2.4. Definitions

CAD is characterized by a ≥ 50% narrowing of the lumen of one main coronary artery, and the severity of CAD depends on the degree of stenosis of the narrowed coronary artery. The Gensini Score is used for a quantitative assessment of coronary artery lesion severity: luminal narrowing ≤ 25% scores 1 point, 25% to 50% scores 2 points, 51% to 75% scores 4 points, 76% to 90% scores 8 points, 91% to 99% scores 16 points, and 100% scores 32 points. These scores are then multiplied by the coefficient of the affected vessel: 5 for the left main artery, 2.5 for the proximal and circumflex branches of the left anterior descending artery, 1.5 for the mid portion of the left anterior descending artery, 1 for the distal left anterior descending artery, obtuse marginal branches, and right coronary artery, and 0.5 for other branches. The total score for CAD in each patient is the sum of scores for all vessel branches, with a higher score indicating more severe stenosis.^[11]^ Patients are categorized into 4 groups based on the Gensini Score quartiles. BMI is calculated using height and weight data, with the formula being weight divided by height squared (weight in kilograms, height in meters). The diagnostic criteria for DM according to the World Health Organization include FPG ≥ 7.0 mmol/L, 2-hour blood glucose level ≥ 11.1 mmol/L based on an oral glucose tolerance test, HbA1c ≥ 6.5%, or a history of type 2 diabetes mellitus. Normal glucose regulation (NGR) is defined as FPG < 6.1 mmol/L and 2-hour blood glucose level < 7.8 mmol/L.^[12]^ Individuals with elevated blood glucose levels but not meeting the diagnostic criteria for DM should be considered as having Pre-diabetes (pre-DM).

### 2.5. Statistical analysis

Based on the CAR values calculated at admission, patients were stratified into quartiles. Quantitative variables were expressed using descriptive statistics comprising arithmetic means with their corresponding standard deviations. Differences in continuous variables between groups were determined using t-tests or Mann-Whitney tests, depending on the normality of the variables. The Kolmogorov-Smirnov test was employed to assess normal distribution. Categorical variables were summarized as percentages and compared using chi-square tests. All statistical analyses were conducted using R software (version 4.2.2) and SPSS 24.0 (IBM Corp, New York), with statistical significance defined as 2-tailed P values < 0.05.

Univariate and multivariate regression analyses were performed to verify the correlation between CAR and the incidence of CAD in the entire population, calculating odds ratios (ORs) and corresponding 95% confidence intervals (CIs). In the multivariate logistic regression analysis, potential confounding factors were adjusted, primarily baseline characteristics (i.e., age, sex, BMI, smoking, hypertension and medication history) with P-values < 0.05, constructing 4 logistic regression models: Model 1, unadjusted; Model 2, adjusted for age, sex, BMI; Model 3, adjusted for age, sex, BMI, SBP, DBP, smoking, alcohol consumption, antihypertensive drug use, and antihypertensive drug use; Model 4 based on Model C adjusted for white blood cell, total cholesterol, triglyceride. Within CAR quartiles, stratified analyses were conducted according to glucose metabolism status. Logistic regression analysis was again used to carefully examine the relationship between CAR and the severity of new-onset CAD. Additionally, we quantitatively assessed the diagnostic efficacy of this biomarker in detecting coronary artery disease through receiver operating characteristic (ROC) analysis. The primary metric for evaluating predictive performance was the area under the curve (AUC).

## 3. Results

### 3.1. Baseline characteristics

The demographic and clinical profiles of the study cohort are presented in Table 1. Our analysis included 1489 eligible subjects with a mean age of 59 years and a male predominance of 71.7% (n = 1067). Among them, 1143 patients (76.8%) had CAD. Based on the quartiles of CAR, participants were divided into 4 groups: Q1 (CAR < 0.026), Q2 (0.026 ≤ CAR < 0.069), Q3 (0.069 ≤ CAR < 0.226), and Q4 (CAR ≥ 0.226). There were significant differences in the incidence and severity of CAD among the groups (*P* < .001). Compared to the Q1 group, patients in the Q4 group had a higher prevalence of CAD. They were more likely to be male and had lower levels of total cholesterol, triglyceride, and HDL-C.

**Table 1:** Characteristics of participants according to CAR quartile.

Characteristic	Total (N = 1489)	CAR quantile				*P*-value
		Q1, N = 372	Q2, N = 372	Q3, N = 372	Q4, N = 373	
Sex, n(%)						.013
Male	1067 (71.7)	250 (67.2)	264 (71.0)	263 (70.7)	290 (77.7)	
Female	422 (28.3)	122 (32.8)	108 (29.0)	109 (29.3)	83 (22.3)	
Age (y)						
Total, median (IQR)	59 (53,67)	59 (52,67)	59 (53,66)	58 (53,66)	60 (52,68)	.091
<60, n (%)	810 (54.4)	209 (56.2)	210 (56.5)	217 (58.3)	174 (46.6)	.006
>60, n (%)	679 (45.6)	163 (43.8)	162 (43.5)	155 (41.7)	199 (53.4)	
SBP, mm Hg, median (IQR)	133 (119,147)	135 (120,147)	136 (120,150)	132 (119,146)	131 (117,146)	.056
DBP, mm Hg, median (IQR)	83 (74,92)	83 (75,91)	83 (76,92)	83 (73,92)	81 (73,93)	.219
TC, mmol/L, median (IQR)	4.47 (3.80,5.26)	4.33 (3.73,5.12)	4.51 (3.89,5.35)	4.70 (3.95,5.32)	4.41 (3.62,5.21)	.003
TG, mmol/L, median (IQR)	1.47 (1.05,2.18)	1.36 (0.96,1.98)	1.67 (1.15,2.47)	1.61 (1.14,2.39)	1.32 (1.00,1.96)	<.001
HDL-C, mmol/L, median (IQR)	1.06 (0.89,1.27)	1.16 (0.98,1.38)	1.03 (0.88,1.25)	1.01 (0.86,1.22)	1.01 (0.86,1.23)	<.001
LDL-C, mmol/L, median (IQR)	2.97 (2.38,3.70)	2.88 (2.27,3.54)	3.02 (2.43,3.73)	3.12 (2.50,3.71)	2.85 (2.33,3.71)	.005
CRP, nmol/L, median (IQR)	3 (1,9)	1 (0,1)	2 (1,2)	5 (4,7)	22 (14,41)	<.001
ALB, g/dL, median (IQR)	41.1 (38.5,43.7)	42.3 (40.3,44.5)	41.7 (39.5,44.3)	40.7 (38.3,43.1)	38.9 (36.1,41.9)	<.001
CAR	0.07 (0.03,0.23)	0.01 (0.01,0.02)	0.04 (0.03,0.05)	0.12 (0.09,0.16)	0.58 (0.35,1.07)	<.001
Smoking, n (%)	771 (51.8)	173 (46.5)	187 (50.3)	197 (53.0)	214 (57.4)	.025
Drinking, n (%)	238 (16.0)	50 (13.4)	59 (15.9)	51 (13.7)	78 (20.9)	.019
Use of antihypertensive, n (%)	595 (40.0)	144 (38.7)	157 (42.2)	146 (39.2)	148 (39.7)	.774
Use of antiplatelet, n (%)	117 (7.9)	24 (6.5)	34 (9.1)	30 (8.1)	29 (7.8)	.596
CAD, n (%)	1143 (76.8)	245 (65.9)	273 (73.4)	298 (80.1)	327 (87.7)	<.001
GS quantile, n (%)						<.001
I	361 (24.2)	134 (36.0)	98 (26.3)	75 (20.2)	54 (14.5)	
II	379 (25.5)	101 (27.2)	111 (29.8)	86 (23.1)	81 (21.7)	
III	372 (25.0)	77 (20.7)	81 (21.8)	108 (29.0)	106 (28.4)	
IV	377 (25.3%)	60 (16.1%)	82 (22.0%)	103 (27.7%)	132 (35.4%)	

ALB = albumin, CAD = Coronary artery disease, CAR = C-reactive protein/albumin ratio, CRP = C-reactive protein, DBP = diastolic blood pressure, GS = gensini score, HDL-C = high-density lipoprotein cholesterol, LDL-C = low-density lipoprotein cholesterol, SBP = systolic blood pressure, TC = total cholesterol, TG = triglyceride, *P* < .05 (two-sided) was defined as statistically significant.

### 3.2. Relationship between the CAR and the risk of new-onset CAD in different glucose metabolic states

We first analyzed the relationship between CRP and CAD. As shown in Table 2, CAR was significantly associated with an increased risk of CAD across all statistical models. In the fully adjusted model (Model 4), the highest CAR quartile (Q4) exhibited a 2.58-fold elevated CAD risk compared to the reference quartile (Q1). A dose-response relationship was observed, with progressively higher ORs from Q2 to Q4. Each standard deviation (SD) increase in CAR conferred a 44% higher CAD risk. Notably, CAR demonstrated stronger effect power for CAD than its components. While CRP was positively associated with CAD, and ALB was protective, their effect sizes were substantially smaller than CAR.

**Table 2:** The relationship of CAR with new-onset CAD risk.

Variables	CAD							
	OR (95%CI)*	*P*-value	OR (95%CI)†	*P*-value	OR (95%CI)‡	*P*-value	OR (95%CI)§	*P*-value
CRP	1.02 (1.01–1.04)	<.001	1.02 (1.01–1.03)	<.001	1.02 (1.01–1.03)	<.001	1.02 (1.01–1.03)	<.001
Per SD increase	1.70 (1.34–2.16)	<.001	1.63 (1.29–2.07)	<.001	1.63 (1.29–2.06)	<.001	1.46 (1.16–1.84)	.003
ALB	0.94 (0.91–0.97)	<.001	0.96 (0.93–0.99)	.027	0.97 (0.93–0.99)	0.046	0.96 (0.92–0.99)	.019
Per SD increase	0.79 (0.69–0.89)	<.001	0.86 (0.75–0.98)	.027	0.87 (0.76–0.99)	0.046	0.84 (0.73–0.97)	.019
CRP/ALB	2.60 (1.69–4.00)	<.001	2.38 (1.56–3.63)	<.001	2.36 (1.55–3.60)	<.001	1.88 (1.24–2.83)	.003
Q1	Reference		Reference		Reference		Reference	
Q2	1.43 (1.04–1.96)	0.026	1.46 (1.04–2.03)	0.027	1.44 (1.03–2.02)	0.035	1.18 (0.83–1.67)	.357
Q3	2.09 (1.50–2.91)	<.001	2.35 (1.65–3.36)	<.001	2.33 (1.63–3.35)	<.001	1.79 (1.23–2.61)	.002
Q4	3.68 (2.53–5.37)	<.001	3.53 (2.38–5.23)	<.001	3.61 (2.42–5.38)	<.001	2.58 (1.69–3.93)	<.001
Per SD increase	1.74 (1.36–2.23)	<.001	1.65 (1.29–2.11)	<.001	1.65 (1.29–2.11)	<.001	1.44 (1.13–1.83)	.003
*P*-trend		<.001		<.001		<.001		<.001

* Model1: unadjusted.

† Model2: adjusted for sex, age, BMI.

‡ Model3: adjusted for sex, age, BMI, SBP, DBP, smoking, drinking, use of antihypertensives, use of antiplatelet.

§ Model4: adjusted for sex, age, BMI, SBP, DBP, smoking, drinking, use of antihypertensives, use of antiplatelet diabetes, WBC, TC, TG.

We further analyzed the relationship between CAR and new-onset CAD risks stratified by glucose metabolism in detail, as shown in Table 3. The results showed that CAR exhibited a strong association with CAD in NGR individuals. In model 4 (fully adjusted), each SD increase in CAR was linked to a 2.08-fold higher CAD risk (odds ratio [OR] = 2.08, *P* < .05). The risk of participants in the highest quartile (Q4) of CAR was 4.25 times higher than that in the lowest quartile (Q1) (OR = 4.25, *P* < .05). In Pre-DM, CAR was still closely related to CAD, though statistical significance was attenuated after full adjustment. It can be seen that in model 4, the risk of CAD in the Q4 group was 2.75 times that in the Q1 group (OR = 2.75, *P* < .05). Significant trends were observed across quartiles (*P*-trend < .05). Among DM patients, the highest CAR quartile (Q4) was associated with a 2.29 times elevated CAD risk (OR = 2.29, *P* < .05). Each SD increase in CAR increased risk by 47% (OR = 1.47, *P* < .05).

**Table 3:** Relationship between the CAR and the risk of new-onset CAD Risk in different glucose metabolic states.

Glucose regulation state	Variables	CAD							
		OR (95%CI)*	*P*-value	OR (95%CI)†	*P*-value	OR (95%CI)‡	*P*-value	OR (95%CI)§	*P*-value
NGR	CAR	6.27 (1.61–24.43)	.008	6.29 (1.38–28.60)	.017	5.66 (1.21–26.50)	.028	6.03 (1.12–32.41)	.036
	Q1	Reference		Reference		Reference		Reference	
	Q2	1.76 (0.86–3.62)	.121	1.96 (0.89–4.31)	.095	1.87 (0.83–4.21)	.128	1.53 (0.66–3.54)	.316
	Q3	1.90 (0.92–3.91)	.083	2.16 (0.96–4.90)	.064	2.02 (0.86–4.73)	.106	1.41 (0.57–3.47)	.452
	Q4	4.25 (1.88–9.60)	<.001	4.99 (1.99–12.56)	<.001	4.80 (1.85–12.43)	.001	4.25 (1.55–11.63)	.005
	Per SD increase	2.11 (1.21–3.68)	.008	2.12 (1.14–3.93)	.017	2.03 (1.08–3.81)	.028	2.08 (1.05–4.13)	.036
	*P*-trend	2.08 (1.21–3.56)	.008	2.23 (1.22–4.07)	.009	2.16 (1.16–4.03)	.015	1.84 (0.96–3.52)	.068
Pre-DM	CAR	2.17 (1.15–4.10)	.017	1.93 (1.03–3.60)	.040	1.90 (1.02–3.54)	.044	1.66 (0.87–3.14)	.124
	Q1	Reference		Reference		Reference		Reference	
	Q2	1.42 (0.88–2.28)	.149	1.40 (0.83–2.36)	.205	1.40 (0.82–2.40)	.218	1.21 (0.69–2.09)	.506
	Q3	2.50 (1.49–4.18)	<.001	3.00 (1.70–5.30)	<.001	3.05 (1.71–5.45)	<.001	2.39 (1.30–4.36)	.005
	Q4	3.47 (2.00–6.01)	<.001	3.35 (1.85–6.07)	<.001	3.40 (1.85–6.25)	<.001	2.75 (1.46–5.19)	.002
	Per SD increase	1.44 (1.07–1.95)	.017	1.36 (1.01–1.84)	.040	1.35 (1.01–1.82)	.044	1.27 (0.94–1.72)	.124
	*P*-trend	2.46 (1.69–3.60)	<.001	2.68 (1.77–4.05)	<.001	2.72 (1.78–4.16)	<.001	2.32 (1.49–3.60)	<.001
DM	CAR	1.99 (1.13–3.53)	.018	1.87 (1.06–3.28)	.030	1.95 (1.10–3.46)	.023	1.72 (1.01–2.97)	.050
	Q1	Reference		Reference		Reference		Reference	
	Q2	1.26 (0.72–2.18)	.417	1.36 (0.77–2.41)	.286	1.37 (0.77–2.45)	.284	1.21 (0.67–2.19)	.521
	Q3	1.26 (0.72–2.18)	.417	1.30 (0.74–2.30)	.360	1.31 (0.74–2.34)	.353	1.12 (0.62–2.02)	.717
	Q4	2.94 (1.51–5.72)	.002	2.76 (1.40–5.45)	.003	2.97 (1.49–5.93)	.002	2.29 (1.12–4.71)	.024
	Per SD increase	1.63 (1.09–2.46)	.018	1.56 (1.04–2.33)	.030	1.61 (1.07–2.42)	.023	1.47 (1.02–2.17)	.049
	*P*-trend	1.62 (1.06–2.47)	.026	1.53 (0.99–2.36)	.055	1.58 (1.02–2.46)	.041	1.35 (0.86–2.13)	.197

DM = diabetes mellitus, NGR = Normal glucose regulation, Pre-DM = Pre-diabetes.

* Model1: unadjusted.

† Model2: adjusted for sex, age, BMI.

‡ Model3: adjusted for sex, age, BMI, SBP, DBP, smoking, drinking, use of antihypertensives, use of antiplatelet.

§ Model4: adjusted for sex, age, BMI, SBP, DBP, smoking, drinking, use of antihypertensives, use of antiplatelet, WBC, TC, TG.

### 3.3. Relationship between CAR and the severity of coronary artery lesion in different states of glucose regulation

In the overall population, as presented in Table 4, in multivariable logistic regression models adjusted for age, sex, etc, CAR exhibited a noticeable correlation with coronary artery severity (OR = 1.38, *P* < .01). Moreover, we described in detail the relationship between CAR stratified by glucose metabolism and the severity of CAD in Table 5. In NGR, while CAR was associated with CAD severity in initial models, significance attenuated after full adjustment. Among Pre-DM and DM patients, CAR consistently predicted CAD severity, even after full adjustment. This correlation appeared to be more pronounced in DM (OR = 1.31, *P* < .01). In the fully adjusted analysis with CAR as a categorical variable, the risk of CAD in the Q3 and Q4 groups of Pre-DM patients was 1.85 times (*P* < .01) and 2.00 times (*P* < .01) higher than that of patients in the Q1 group compared with the Q1 group, and the risk of CAD in the Q3 and Q4 groups of DM was 1.55 times (*P* < .05) and 2.51 times (*P* < .001).

**Table 4:** Relationship between the CAR with new-onset CAD severity.

Variables	CAD							
	OR (95%CI)*	*P*-value	OR (95%CI)†	*P*-value	OR (95%CI)‡	*P*-value	OR (95%CI)§	*P*-value
CAR	1.63 (1.37–1.94)	<.001	1.59 (1.34–1.90)	<.001	1.59 (1.33–1.90)	<.001	1.38 (1.16–1.66)	<.001
Q1	Reference		Reference		Reference		Reference	
Q2	1.46 (1.13–1.90)	.004	1.50 (1.16–1.95)	.002	1.49 (1.14–1.93)	0.003	1.24 (0.94–1.62)	.123
Q3	2.19 (1.69–2.85)	<.001	2.36 (1.81–3.07)	<.001	2.32 (1.78–3.02)	<.001	1.82 (1.39–2.39)	<.001
Q4	3.07 (2.36–3.99)	<.001	2.95 (2.26–3.85)	<.001	2.92 (2.23–3.82)	<.001	2.13 (1.61–2.82)	<.001

* Model1: unadjusted.

† Model2: adjusted for sex, age, BMI.

‡ Model3: adjusted for sex, age, BMI, SBP, DBP, smoking, drinking, use of antihypertensives, use of antiplatelet.

§ Model4: adjusted for sex, age, BMI, SBP, DBP, smoking, drinking, use of antihypertensives, use of antiplatelet diabetes, WBC, TC, TG.

**Table 5:** Relationship between the CAR with new-onset CAD severity in different states of glucose regulation.

Glucose regulation state	Variables	CAD							
		OR (95%CI)*	*P*-value	OR (95%CI)†	*P*-value	OR (95%CI)‡	*P*-value	OR (95%CI)§	*P*-value
NGR	CAR	1.99 (1.14–3.47)	.016	1.81 (1.03–3.17)	.038	1.66 (0.94–2.94)	.080	1.26 (0.69–2.30)	.443
	Q1	Reference		Reference		Reference		Reference	
	Q2	1.94 (1.04–3.63)	.037	1.98 (1.05–3.76)	.036	1.93 (1.01–3.69)	.047	1.51 (0.78–2.94)	.223
	Q3	2.69 (1.40–5.17)	.003	2.82 (1.44–5.51)	.003	2.59 (1.31–5.13)	.006	1.70 (0.83–3.47)	.147
	Q4	3.04 (1.62–5.70)	<.001	2.84 (1.49–5.41)	.002	2.66 (1.38–5.15)	.004	1.91 (0.96–3.80)	.064
Pre-DM	CAR	1.50 (1.07–2.10)	.018	1.47 (1.04–2.07)	.029	1.45 (1.03–2.04)	.035	1.27 (1.01–1.82)	.048
	Q1	Reference		Reference		Reference		Reference	
	Q2	1.54 (1.03–2.32)	.037	1.57 (1.04–2.37)	.033	1.62 (1.07–2.47)	.023	1.44 (0.94–2.20)	.090
	Q3	2.12 (1.42–3.17)	<.001	2.20 (1.46–3.31)	<.001	2.19 (1.45–3.30)	<.001	1.85 (1.21–2.82)	.004
	Q4	2.58 (1.72–3.86)	<.001	2.44 (1.62–3.68)	<.001	2.40 (1.59–3.62)	<.001	2.00 (1.31–3.07)	.001
DM	CAR	1.45 (1.19–1.77)	<.001	1.39 (1.13–1.69)	.001	1.38 (1.12–1.69)	.002	1.31 (1.06–1.61)	.012
	Q1	Reference		Reference		Reference		Reference	
	Q2	1.29 (0.86–1.92)	.222	1.39 (0.92–2.09)	.117	1.32 (0.87–1.99)	.192	1.16 (0.76–1.77)	.482
	Q3	1.79 (1.19–2.68)	.005	1.90 (1.26–2.85)	.002	1.79 (1.18–2.70)	.006	1.55 (1.02–2.37)	.040
	Q4	3.27 (2.17–4.91)	<.001	3. 33 (2.20–4.81)	<.001	3.09 (2.03–4.70)	<.001	2.51 (1.62–3.89)	<.001

DM = diabetes mellitus, NGR = Normal glucose regulation, Pre-DM = Pre-diabetes.

* Model1: unadjusted.

† Model2: adjusted for sex, age, BMI.

‡ Model3: adjusted for sex, age, BMI, SBP, DBP, smoking, drinking, use of antihypertensives, use of antiplatelet.

§ Model4: adjusted for sex, age, BMI, SBP, DBP, smoking, drinking, use of antihypertensives, use of antiplatelet, WBC, TC, TG.

### 3.4. Predictive performance of CRP in CAD in different states of glucose regulation

**Figure 2.**
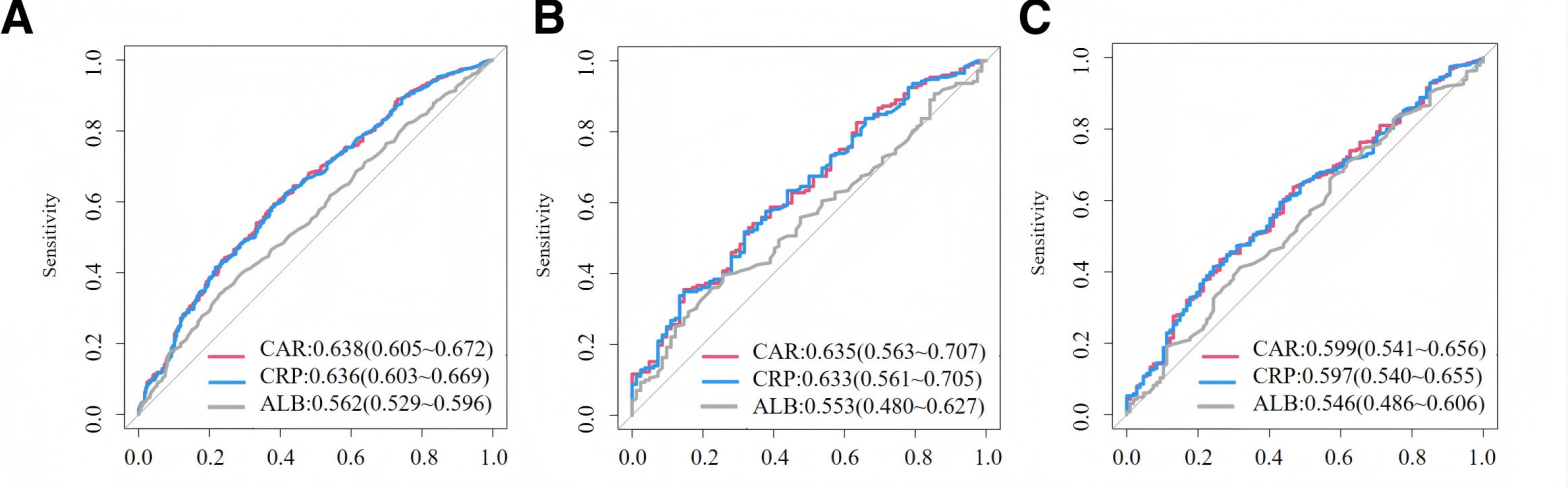
ROC curve analysis of the CAR for CAD prediction (A) ROC curve analysis of the CRP, ALB, and CAR for CAD prediction in the total population. (B) ROC Curves of the CRP, ALB, and CAR for CAD prediction in normal glucose regulation. (C) ROC Curves for CRP, ALB, and CAR for CAD prediction in diabetes mellitus.

ROC curve analyses evaluating the predictive ability of CRP, ALB, and CAR for CAD are presented in Figure 2. The analysis within the total population (Fig. 2A) demonstrated that CAR had an AUC of 0.638 (95% confidence interval [CI]: 0.605–0.672) for predicting CAD. The optimal cutoff value for diagnosing CAD was 0.059. Compared with CRP or ALB alone, the AUC of CAR is slightly higher, demonstrating better predictive performance. Furthermore, this predictive advantage of CAR still exists after stratification based on glucose metabolic status (Fig. 2B–C).

## 4. Discussion

CAD is the leading cause of death worldwide, and aging exacerbates its burden even further.^[13,14]^ Although traditional risk factor models, such as SCORE2, have been widely used, the pathogenesis of CAD is highly heterogeneous, which limits the accuracy of individual risk prediction.^[15]^ Previous studies have suggested a close association between CAD and inflammation, glucose metabolism, and nutrition; however, the specific relationship remains unclear.^[16,17]^ The focus of this study was the relationship between CAR and new-onset coronary heart disease, as well as the severity of the disease, under different glucose metabolism states. The main findings are as follows: First, CAR emerged as an independent predictor of new-onset CAD in both NGR and DM cohorts. However, this predictive capacity was absent in Pre-DM individuals. Second, a robust correlation between CAR and CAD severity was observed in both Pre-DM and DM groups. The most pronounced effect was observed in DM patients. Third, CAR demonstrated superior predictive capability over individual CRP or albumin measures. This establishes CAR as a promising biomarker for newly diagnosed CAD.

The interaction between glucose status and CAD is complex and involves inflammatory and nutritional pathways. Inflammation is a key factor in initiating and amplifying systemic insulin resistance (IR), and it is one of the important driving forces in the pathogenesis of T2DM.^[18,19]^ Chronic low-grade inflammation can promote endothelial dysfunction, dyslipidemia, and myocardial cell damage. Ultimately, it accelerates the formation of atherosclerotic plaques, which is the primary mechanism of coronary heart disease in diabetic patients.^[20]^ IR, the key pathophysiological driver of type 2 diabetes, directly promotes hepatic CRP synthesis via pro-inflammatory cytokines such as interleukin-6 and tumor necrosis factor-a.^[21]^ Simultaneously, IR-induced oxidative stress and endothelial dysfunction impair albumin production and accelerate its catabolism, leading to hypoalbuminemia.^[22]^ The CAR therefore captures both the pro-inflammatory and the metabolic-nutritional consequences of IR in a single composite metric. As IR progresses from NGR to Pre-DM and then to DM, CAR levels rise accordingly. Meanwhile, hypoalbuminemia reflects nutritional impairment and systemic inflammation. Reduced albumin levels are independently associated with coronary heart disease mortality and diabetic microvascular complications.^[6,23]^ Given this interaction, accurately assessing inflammatory and nutritional status is critical for cardiovascular risk stratification, especially in the context of abnormal glycemic status. Bayrak et al reported that elevated CAR in diabetes is highly associated with coronary heart disease,^[24]^ suggesting that CAR may bridge metabolic disorders in diabetes and the pathogenesis of CAD. In our study, we included the composite index CAR, which assesses inflammation and nutritional status, to evaluate the relationship between glucose metabolism status and new-onset CAD patients.

This study examined the relationship between CAR and new-onset CAD under different glucose metabolism states, revealing that CAR’s ability to predict coronary heart disease depends on metabolism. While Tanriverdi et al indicated that CAR had the strongest diagnostic value in detecting significant CAD^[25]^ and Akkaya et al identified CAR as a predictor of multi-vascular CAD,^[26]^ neither study focused on the impact of glucose metabolism on CAR’s predictive ability for CAD. Our research addresses this gap, showing that CAR can predict CAD in the NGR and DM groups, though this association is not apparent in Pre-DM. This difference may depend on pathophysiological dynamic changes during the progression of abnormal blood glucose. In Pre-DM, adaptive compensatory responses, such as the high insulin-induced anti-inflammatory effects,^[27]^ may weaken the connection between CAR and CAD. Moreover, the degree of inflammation and metabolic disorders in individuals with Pre-DM is relatively mild and dynamic. The body may be in an adaptive compensatory state, not yet reaching an imbalance sufficient to significantly affect CRP and ALB levels. This makes CAR unable to effectively predict coronary heart disease risk.^[28]^ In DM, persistent hyperglycemia amplifies systemic inflammation through advanced glycation end products and oxidative stress.^[29]^ Related metabolic disorders may lead to the impairment of albumin’s structure and function,^[30]^ which reduces its protective effect on blood vessels and enhances CAR’s predictive ability.

Our research further explored the relationship between CRP and the severity of coronary heart disease. Previous studies have observed that STEMI patients with proximal or midcourse lesions have higher CAR levels than those with distal lesions.^[31]^ Additionally, LOU et al found that the CAR is positively correlated with the severity of coronary heart disease.^[32]^ However, few studies have examined the impact of different blood glucose states on the degree of coronary artery disease. This study conducted a stratified analysis and found that CAR significantly associates with the severity of coronary artery disease throughout abnormal blood glucose states, particularly in DM patients. During this stage of abnormal blood glucose, chronic hyperglycemia stimulates the liver to produce excessive amounts of CRP by activating IL-6.^[29]^ CRP then promotes endothelial dysfunction by activating the LOX-1 receptor^[33]^ and simultaneously inhibits ALB synthesis by inducing endoplasmic reticulum stress.^[34]^ CAR uniquely reflects this dual pathology. Elevated CRP directly promotes endothelial dysfunction and plaque instability, while hypoalbuminemia weakens vascular protection by reducing nitric oxide’s bioavailability and antioxidant capacity.^[35,36]^ Meanwhile, both CRP and albumin can regulate platelet aggregation and fibrinolysis,^[26,37]^ and their combined effect ultimately accelerates atherosclerosis. In conclusion, our results enhance understanding of the interaction between inflammation and CAD under different glycemic states. Our findings build on the emphasis placed by other researchers on the role of inflammation in CAD development.^[38,39]^ In clinical practice, CAR’s accessibility and cost-effectiveness make it a promising tool for risk stratification in patients with different blood glucose statuses and coronary heart disease.

However, it remains controversial whether CAR can be beneficial for predicting CAD compared with CRP and ALB alone. One retrospective study of 815 patients have ST-segment elevation myocardial infarction reported that CAR was more closely correlated with the complexity and severity of CAD than CRP and ALB alone.^[40]^ Conversely, another prospective study of 411,506 patients showed that CAR was no better than CRP at predicting mortality or cardiovascular disease.^[41]^ These contradictory results may be due to the selection of the included patients. Therefore, to avoid prevalence-incidence bias, we selected patients with newly diagnosed coronary heart disease as our research subjects. This study used the ROC curve to compare the differences among the 3 biomarkers, confirming that CAR’s predictive ability for CAD was slightly higher than CRP’s or albumin’s alone. This finding was consistent in cohorts of patients with normal blood glucose levels and diabetes. The enhanced predictive ability may stem from CAR’s inherent role as a comprehensive measure of persistent inflammation and nutritional status. The study suggests the potential utility of CAR in evaluating the severity and prognosis of CAD.

## 5. Strengths and limitations

This study’s advantage lies in being the first dedicated investigation evaluating the impact of CAR on the risk of new-onset CAD in different glucose metabolism states. The patients included were undergoing CAG for the first time and were new-onset CAD, which helped in avoiding potential biases from long-term use of CAD secondary prevention medications as well as preventing disease incidence-prevalence bias. The study had a large sample size, and multiple confounding factor models were established and adjusted, yielding convincing results. These results provide valuable insights but should be viewed with consideration of their limitations. Firstly, as a cross-sectional study, with CAR determined based on baseline data, this study lacks temporal data required for establishing causal relationships. Having dynamic data could enhance the value of stratifying CAD risk. Secondly, the potential impact of long-term use of antihypertensive, antidiabetic, and antilipid medications on lipid and glucose levels measurements, as well as CAD incidence, cannot be ruled out. Thirdly, the single-institution design and exclusive focus on Chinese participants introduce a selection bias, and the findings may not apply to a wider population. Future research should consider these factors to further enhance the accuracy of study outcomes.

## 6. Conclusion

CAR demonstrates robust and independent correlations with CAD risk and coronary lesion severity, outperforming isolated CRP or albumin metrics in CAD prediction. Critically, the CAR-CAD incidence association exhibits glycometabolic status dependency, underscoring CAR’s promise for refining cardiovascular risk stratification. As a clinically accessible biomarker, CAR may enable early identification of high-risk CAD individuals, facilitating precision-based interventions.

## Author contributions

**Data curation:** Sisi Han, Liangbo Chen, Hongyun Shu.

**Investigation:** Liangbo Chen.

**Methodology:** Sisi Han, Hongyun Shu.

**Project administration:** Liangbo Chen.

**Resources:** Henghui Zhang, Jianhong Li, Guihua Zheng.

**Software:** Weiping Qiu, Jingsong Cheng.

**Validation:** Sisi Han.

**Writing – original draft:** Sisi Han, Liangbo Chen, Hongyun Shu.

**Writing – review & editing:** Guojun Zhao, Qiaowen Li.
